# Red Blood Cell Transfusions in Greece: Results of a Survey of Red Blood Cell Use in 2013

**DOI:** 10.4274/tjh.2016.0188

**Published:** 2017-03-01

**Authors:** Serena Valsami, Elisavet Grouzi, Abraham Pouliakis, Leontini Fountoulaki-Paparisos, Elias Kyriakou, Maria Gavalaki, Elias Markopoulos, Ekaterini Kontopanou, Ioannis Tsolakis, Argyrios Tsantes, Alexandra Tsoka, Anastasia Livada, Vassiliki Rekari, Niki Vgontza, Dimitra Agoritsa, Marianna Politou, Stavros Nousis, Aspasia Argyrou, Ekaterini Manaka, Maria Baka, Maria Mouratidou, Stavroula Tsitlakidou, Konstantinos Malekas, Dimitrios Maltezos, Paraskevi Papadopoulou, Vassiliki Pournara, Ageliki Tirogala, Emmanouil Lysikatos, Sousanna Pefani, Konstantinos Stamoulis

**Affiliations:** 1 On Behalf of the Working Committee of Transfusion Medicine & Apheresis of the Hellenic Society of Hematology

**Keywords:** red blood cell, Transfusion practice, Blood storage age

## Abstract

**Objective:**

Greece is ranked as the second highest consumer of blood components in Europe. For an effective transfusion system and in order to reduce variability of transfusion practice by implementing evidence-based transfusion guidelines it is necessary to study and monitor blood management strategies. Our study was conducted in order to evaluate the use of red blood cell units (RBC-U) in nationwide scale mapping parameters that contribute to their proper management in Greece.

**Materials and Methods:**

The survey was conducted by the Working Committee of Transfusion Medicine&Apheresis of the Hellenic Society of Hematology from January to December 2013. The collected data included the number, ABO/D blood group, patients’ department, and storage age of RBC-U transfused.

**Results:**

The number of RBC-U evaluated was 103,702 (17.77%) out of 583,457 RBC-U transfused in Greece in 2013. RBC-U transfused by hospital department (mean percentage) was as follows: Surgery 29.34%, Internal Medicine 29.48%, Oncology/Hematology 14.65%, Thalassemia 8.87%, Intensive Care Unit 6.55%, Nephrology 1.78%, Obstetrics/Gynecology 1.46%, Neonatal&Pediatric 0.31%, Private Hospitals 8.57%. RBC-U distribution according to ABO/D blood group was: A: 39.02%, B: 12.41%, AB: 5.16%, O: 43.41%, D+: 87.99%, D-: 12.01%. The majority of RBC-U (62.46%) was transfused in the first 15 days of storage, 25.24% at 16 to 28 days, and 12.28% at 29-42 days.

**Conclusion:**

Despite a high intercenter variability in RBC transfusions, surgical and internal medicine patients were the most common groups of patients transfused with an increasing rate for internal medicine patients. The majority of RBC-U were transfused within the first 15 days of storage, which is possibly the consequence of blood supply insufficiency leading to the direct use of fresh blood. Benchmarking transfusion activity may help to decrease the inappropriate use of blood products, reduce the cost of care, and optimize the use of the voluntary donor’s gift.

## Abstract (tr)

**Amaç:**

Avrupa’daki kan bileşenlerini en çok tüketen ülkeler arasında Yunanistan ikinci sıradadır. Etkili bir transfüzyon sistemi için ve transfüzyon uygulamasının değişkenliğini azaltmak için kanıta dayalı transfüzyon kılavuzlarını uygulayarak kan yönetimi stratejilerini incelemek ve izlemek gereklidir. Çalışmamız, Yunanistan’da doğru kan transfüzyon yönetimine katkıda bulunmak için, ülke çapında ölçek eşleştirme parametrelerinde eritrosit süspansiyonu (ES) kullanımını değerlendirmek amacıyla yürütülmüştür.

**Gereç ve Yöntemler:**

Anket, Ocak-Aralık 2013 tarihleri arasında Hellenic Hematoloji topluluğunun, Transfüzyon Tıbbı ve Aferez Çalışma Komitesi tarafından yürütülmüştür. Toplanan veriler, ABO/D kan grubu, hasta bölümleri ve transfüze edilen ES depolama yaşını içermektedir.

**Bulgular:**

2013 yılında Yunanistan’da transfüzyon yapılan 583,457 ES’nin, ES sayısı 103,702 (%17,77) idi. Hastanedeki bölümlerde transfüze edilen ES (ortalama oran) şöyleydi: Cerrahi %29,34, Dahiliye %29,48, Onkoloji/Hematoloji %14,65, Talasemi %8,87, Yoğun bakım ünitesi %6,55, Nefroloji %1,78, Kadın Hastalıkları ve Doğum %1,46, Yenidoğan ve Çocuk bölümü %0,31, Özel Hastaneler %8,57. ABO/D kan grubuna göre ES dağılımıysa şöyleydi: A: %39,02, B: %12,41, AB: %5,16, O: %43,41, D+: %87,99, D-: %12,01. ES’nin çoğunluğu (%62,46) depolamanın ilk 15 günü, 16 ile 28 günleri arası %25,24’ü ve 29 ile 42 gün arasında ise %12,28’i, transfüze edildi.

**Sonuç:**

Eritrosit transfüzyonlarında merkezler arası yüksek değişkenliğe rağmen, en fazla transfüzyon yapılan hasta grubunu cerrahi ve dahili tıp hastaları oluşturmaktaydı, dahili tıp hastalarında transfüzyon oranları daha yüksekti. ES’nin çoğunluğu depolamanın ilk 15 gününde transfüze edildi; bu da muhtemelen kan depolamadaki yetersizliğinin sonucunda taze kan kullanımına yol açtı. Transfüzyon aktivitesinin standartları, kan ürünlerinin uygun olmayan kullanımını azaltmaya, bakım maliyetini düşürmeye ve gönüllü vericilerin hediye kullanımını optimize etmeye yardımcı olabilir.

## INTRODUCTION

Greece is a member of the European Union, which has established guidelines for blood donation and inspection of blood establishments, but so far no uniform rules for treatment with blood and blood products have been adopted by the European Union. Accordingly, Greek authorities and blood donor associations adhere strictly to the principle of self-sufficiency that was laid out by the Council of Europe. The only source of blood in Greece is non-remunerated blood donors. In a blood system based on voluntary donation the potential for blood shortage is an ongoing risk [1]. A number of emergency scenarios, including natural or man-made disasters, pandemic outbreaks, extremes of weather, and seasonal variations of blood donations, could contribute to extremely low blood inventory levels. It seems clear that the proportion of the population eligible to donate blood is likely to fall over the coming decades while the proportion requiring these products is likely to rise. Further attention is therefore required both to manage the supply and influence the demand for existing blood and blood products.

Greece is ranked as the second highest consumer of blood components in Europe. Blood utilization in Greece exceeds 600,000 red blood cell (RBC) units annually according to data provided by the national competent authority (Hellenic National Blood Transfusion Center). Adequate transfusion practice is essential in order to cover transfusion demands. Assessing data regarding RBC units transfused at medical institutions nationally could provide the data needed for developing plans to manage the demand and supply for blood units [2,3,4]. The aim of our study was to assess and evaluate the use of RBC units in Greece in order to identify parameters that contribute to proper RBC management, which can ensure blood sufficiency, taking into account the geographical particularities of our country, the large number of transfusion-dependent thalassemia patients, and the large number of car accident victims.

## MATERIALS AND METHODS

The study was conducted by the Working Committee of Transfusion Medicine&Apheresis of the Hellenic Society of Hematology. A preprinted data collection form was used and all transfusion services in hospitals all over Greece were invited to participate in the study. The survey was conducted from January to December 2013. Data collection was prospective, using preprinted forms that were filled out monthly by the participating transfusionists. Monthly collected data included the number of RBC units transfused, the ABO/D blood group, and the departments of the patients who received the RBC units. According to storage age (SA) on the day of transfusion the RBC units were sorted into groups as SA1: 0-15 days (SA on the day of transfusion), SA2: 16-28 days, and SA3: 29-42 days [5,6]. Data regarding national RBC transfusion supplies were provided by the Hellenic National Blood Transfusion Center.

Data forms were manually entered into an electronic database (Excel 2007, Microsoft Corp., Redmond, WA, USA), which was also used to perform part of the analysis. Additional statistical analysis was performed using SAS software (version 9.3 for Windows, SAS Institute Inc., Cary, NC, USA) [7,8]. Proportion comparisons were performed via the Z-test, and mean values were compared via the t-test, the accepted significance level was p<0.05.

## RESULTS

From among the 94 services initially invited, transfusion services in 23 hospitals all over Greece accepted the invitation and were eligible to participate in the study. Twelve of those 23 hospitals are located in Athens and the remaining 11 were general hospitals located in cities outside of Athens (Agrinio, Messologgi, Kavala, Zakynthos, Kefalonia, Livadia, Trikala, Larissa, Edessa, Xanthi, Florina) (Table 1). Thirteen of the 23 hospitals (56.52%) provided data for 12 months, 9 hospitals (39.13%) for 5-8 months, and one hospital (4.35%) for 1 month. The mean number of monthly reports from the participating blood banks was 9.2±3.5 and this showed a declining trend over the course of the year (20 reports were received in January 2013, while 15 reports were received in December 2013). It is worth noting that participating hospitals were sending their reports on a voluntary basis.

The total number of RBC units evaluated was 103,702 out of 583,457 [103,702/583,457=17.77±0.10%, 95% confidence interval (CI)] RBC units transfused during 2013 in Greece. The number of units reported by the 12 hospitals in Athens was 76,068 (73.35±0.29%, 95% CI) while the units reported by the 11 hospitals outside of Athens was 27,634 (26.65±0.27%, 95% CI).

More than 64% (66,293/103,702, ±0.29%, 95% CI) of the total RBC units were transfused at five tertiary and general hospitals, four of which are located in Athens and account for 55.72±0.30% (57,784/103,702, 95% CI) of the annual blood issued, along with one hospital outside of Athens (University Hospital of Larissa), as shown in Table 1.

**Figure 1:**
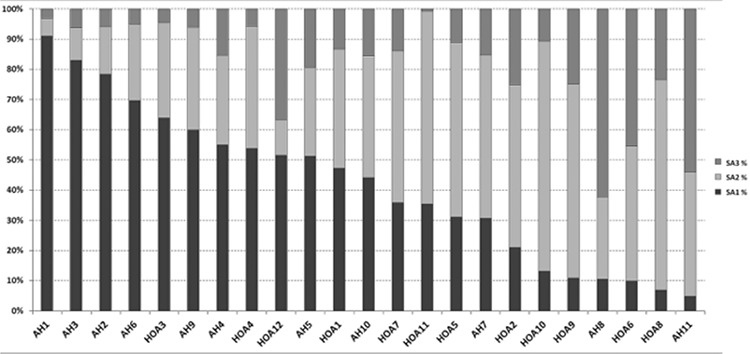
Percentages of red blood cell consumption for the three storage age groups (SA1: 0-15 days, SA2: 16-28 days, and SA3: 29-42 days) for the participating hospitals. Abbreviations: AH: Athens Hospital, HOA: Hospital Outside Athens, AH1: Evangelismos Hospital, AH2: Laikon Hospital, AH3: General Hospital “Saint Panteleimon”, AH4: General Hospital “ATTIKON”, HOA1: Larissa University Hospivtal, AH5: St. Savvas Oncology Hospital, HOA2: General Hospital of Xanthi, AH6: General Hospital Nea Ionia “Agia Olga”, HOA3: General Hospital of Trikala, AH7: Thriasio Hospital, AH8: Aretaeio University Hospital, HOA4: General Hospital Edessa, AH9: “Amalia Fleming” Hospital, AH10: “Sotiria” Hospital, AH11: “Saints Anargyroi” Hospital, HOA5: General Hospital of Kavala, HOA6: General Hospital of Agrinio, HOA7: General Hospital of Messologgi, HOA8: General Hospital of Livadia, HOA9: General Hospital of Florina, HOA10: General Hospital of Zakynthos, HOA11: General Hospital of Kalymnos, HOA12: General Hospital of Kefalonia.

The percentage of RBC units in each SA group (SA1, SA2, and SA3) varied depending on the participating hospital (SA1: 4.94%-91.08%, SA2: 5.83%-76.01%, SA3: 0.74%-45.49%), as shown in Table 2 and Figure 1. The majority of RBC units, 64,799 (62.46±0.29%, 95% CI), were transfused in the first 15 days of storage, while 26,171 (25.24±0.26%, 95% CI) were transferred at 16 to 28 days and 12,732 (12.28±0.20%, 95% CI) were transferred at 29 to 42 days (Table 2).

In order to investigate the different policies applied in large tertiary university hospitals located in urban centers and peripheral, small non-university hospitals (100-300 beds), two groups were created: university urban hospitals (AH1, AH2, and AH4) and general peripheral hospitals (HOA2, HOA3, HOA4, HOA5, HOA6, HOA7, and HAO9). The number of units transfused in urban university hospitals was 44,427 and in peripheral hospitals it was 14,601. Interestingly, university hospitals consumed “fresher” blood compared to peripheral hospitals (SA1 group: 78.9% vs. 38.2%, p<0.05), and accordingly peripheral hospitals used “older” blood (SA3 group: 15.8% vs. 6.8%, p<0.05).

As depicted in Table 2, regarding the total number of RBC units transfused by hospital department, the classification was as follows: Surgery departments: 30,421 (29.34±0.28%, 95% CI), Internal medicine departments: 30,567 (29.48±0.28%, 95% CI), Oncology/Hematology departments: 14,159 (14.65±0.22%, 95% CI), Thalassemia departments: 9195 (8.87±0.17%, 95% CI), Intensive care units: 6796 (6.55±0.15%, 95% CI), Nephrology departments: 1850 (1.78±0.08%, 95% CI) Obstetrics/Gynecology departments: 1512 (1.46±0.07%, 95% CI), Neonatal and Pediatric departments: 319 (0.31±0.03%, 95% CI), and private hospitals: 8883 (8.57±0.17%, 95% CI). It is worth mentioning that significant differences were observed regarding the number of RBC units per department between participating hospitals (data not shown).

The proportion of RBC units transfused in surgery departments of urban university hospitals was greater than that of peripheral hospitals (32.7% vs. 24.2%, p<0.05). A similar pattern was observed in Oncology/Hematology departments (17.6% vs. 0.5%, p<0.05), while in internal medicine departments the percentages were 31.0% and 46.7%, respectively (p<0.05). Regarding thalassemia patients, only 4.4% of RBC units were transfused in urban university hospitals and 17.2% in peripheral general hospitals.

The SA group (SA1, SA2, and SA3) of RBC units transfused by hospital department classification is shown in Table 2 and Figure 1. Neonates and thalassemia patients received “fresh” RBC units of the SA1 group in a higher proportion than patients in the rest of the departments; specifically, 84.95% and 87.31% of cases of neonates and thalassemia patients respectively received SA1 RBC units while the percentage of the total studied population that received SA1 RBC units was 62.49%. This difference was statistically significant both for neonates (difference: 22.46%, 95% CI: 17.623%-26.516%, χ^2^=57.18, p<0.05) and for thalassemia patients (difference: 24.82%, 95% CI: 23.984%-25.632%,χ^2^=1938.95, p<0.05).

The distribution of RBC units transfused according to ABO and RhD blood groups was: A: 40,461 (39.02±0.30%, 95% CI), B: 12,868 (12.41±0.20%, 95% CI), AB: 5355 (5.16±0.13%, 95% CI), O: 45,018 (43.41±0.30%, 95% CI), D (+): 91,248 (87.99±0.20%, 95% CI), D (-): 12,454 (12.01±0.20%, 95% CI). This reflects the ABO/D distribution in the Greek population [9,10].

RBC units per ABO/D blood group and SA group distribution are depicted in Table 3. The distributions among SA1, SA2, and SA3 SA groups were similar for all ABO/D blood groups. In particular, the transfusion practice applied to O RhD-negative blood units was identical to other blood groups, as 63.2% of O RhD-negative units were transfused in the first 2 weeks while the percentage of RBC units of the SA1 group for the rest of the RBC types was 62.4% (difference: 0.76%, p=0.26).

The mean number of RBC units transfused per month in all hospitals was 8642±604 (CI=95%). Monthly distribution of transfusions and SA data, as depicted in Table 4, show that older blood (SA3) was issued during the summer months of May, June, and July. Specifically, 4615 SA3 RBC units were issued during these three months [mean: 1538.3, standard deviation (SD): 349.3], while 8117 SA3 RBC units were issued during the rest of the year (mean: 901.9, SD: 295.6) (p<0.05). Additionally, in terms of consumption, the months of May, June, and July presented increased requirements for transfusions (mean units/month: 9213), while for the rest of the year a mean of 8451 units/month were used, reflecting an increment of about 9% (p<0.05).

## DISCUSSION

Effective blood management is affected not only by donor deficit but also by the complexity of managing inventories of blood products and availability within hospitals and health systems. Overuse or inappropriate use of blood products is a less-recognized problem that presents significant patient safety issues [11,12]. Assessing the RBC transfusion trends in various clinical settings, especially at the national level, has evolved into a major tool for promotion and development of best practices for hemotherapy [2]. In this setting we conducted a benchmark study for RBC use across Greece.

The legal and regulatory framework governing the organization and functioning of Greek blood services reflects the transposition of EU dedicated directives. Attention at the decision-making level focused mainly on strengthening vigilance and the safety of blood supplies [13]. Blood transfusion services in Greece continue to be decentralized, are located in almost every hospital, and are responsible for the whole blood transfusion chain. Blood supplies come from voluntary non-remunerated donors (51%) and replacement donors (49%). Greece has 32 blood donors/1000 inhabitants, which is close to the median range of the EU average [1,14]. The total blood collection figure for 2013 was of 590,000 units and this proved insufficient to cover consumption at the national level, according to data provided by the Hellenic National Blood Transfusion Center. Blood insufficiency in Greece is related not only to increased demands but also to poor implementation of patient blood management programs, and to the fact that central inventory management (i.e. an online system) across the country has not been applied yet.

In our study, data from 23 blood transfusions services regarding 103,702 RBC units transfused during the year 2013 were evaluated. The sample size was considered representative and thus the analysis led to safe conclusions (with a 95% confidence interval, margin of error was 0.28%).

The number of units reported by the 12 hospitals in Athens was 2.75 times greater than the units reported by the 11 hospitals outside Athens (73.35% vs. 26.65%). Interestingly, the majority of RBCs were transfused in the first 15 days of storage (62.49±0.29). In this case, the use of fresh blood possibly highlights the problem of blood sufficiency in our country, which leads to the direct use of fresh blood. Transfusion of blood in the first 15 days of storage (SA1) was a phenomenon more pronounced in hospitals with the highest blood consumption, mainly urban university hospitals (Figure 1). These hospitals have extended Surgical departments also treating multiple-trauma patients as reference centers. However, according to the last census results of 2011, Athens contains 35% of the population of Greece [15]. This reverse percentage in relation to the population is indicative of the fact that health care services focus on the country’s capital. Accordingly, increased consumption of “older” blood (SA3) takes place mainly in small hospitals, including countryside ones, with limited inventory that mostly treat chronic patients. These small hospitals often use RBC units close to the expiry date supplied by other hospitals in order to decrease time expiry losses, according to data provided by the Hellenic National Blood Transfusion Center.

Regarding the total number of RBC units transfused by hospital department and despite intercenter variability, reflecting the existing variability in transfusion practice in our country, the vast majority of RBC units i.e. 75,138 units (73.47±0.27%, 95% CI) were transfused for patients in Surgery and Internal medicine departments, including Hematology/Oncology patients. The lack of strong evidence supporting specific transfusion practices could explain the overuse of blood products in specific patient populations [16,17]. Neonates and thalassemia patients received RBCs of the younger SA group in a statistically significant higher proportion (p<0.05), which has been considered as good transfusion practice by several studies for both patient populations [3,18,19]. Blood consumption in multiple-trauma patients could not be assessed due to the establishment plan of public hospitals in Greece that does not include an independent Accident and Emergency department.

The similar distribution of ABO/D blood groups across RBC units of the three SA groups (Table 3) highlights the lack of an established policy for appropriate use of group O RhD-negative RBC units as in other developed countries. An additional explanation could also be that there has not yet been established a centralized targeted recruitment of O RhD-negative universal donors. Provision of O RhD-negative RBCs can be a challenge for blood services, especially in times of short supply or increased demand [2,20].

According to Table 4, depicting monthly distribution of transfusions and SA data, older blood is issued during summer. May, June, July, and August are the months of summer holidays in Greece, with an impact on RBC stocks due to the decline in blood donation. Consequently, the system reacts by providing stocked RBCs of higher SA groups (SA2 and SA3). In addition, during the summer, many tourists visit Greece. The population increase along with car accident victims results in higher blood transfusion demands. Thus, implementing more intensive voluntary blood donation campaigns could help more intensively to meet the increased demands during these months, as in other developed countries [2].

## CONCLUSION

According to our study, and despite a high intercenter variability in RBC transfusions, surgical and internal medicine patients continue to be the most common group of patients transfused with an increasing rate for internal medicine patients. Additionally, it was revealed that the majority of RBC units were transfused within the first 15 days of storage. The applied blood transfusion trend in our country seems to follow the European practice regarding the transfusion of fresh blood in certain specific patient populations such as neonates and multi-transfused thalassemia patients. However, the increased use of fresh blood possibly reveals the problem of blood sufficiency, which leads to the direct use of fresh blood due to increased demand. The conduction of a larger survey that incorporates the determinants of patient blood management with the geographical particularities related to blood transport difficulties, hospital capacity variation, data regarding RBC wastage, and blood units supplied by other hospitals could provide more data and conclusions needed for developing and implementing an integrated evidence-based transfusion strategy and structure.

## Figures and Tables

**Table 1:**
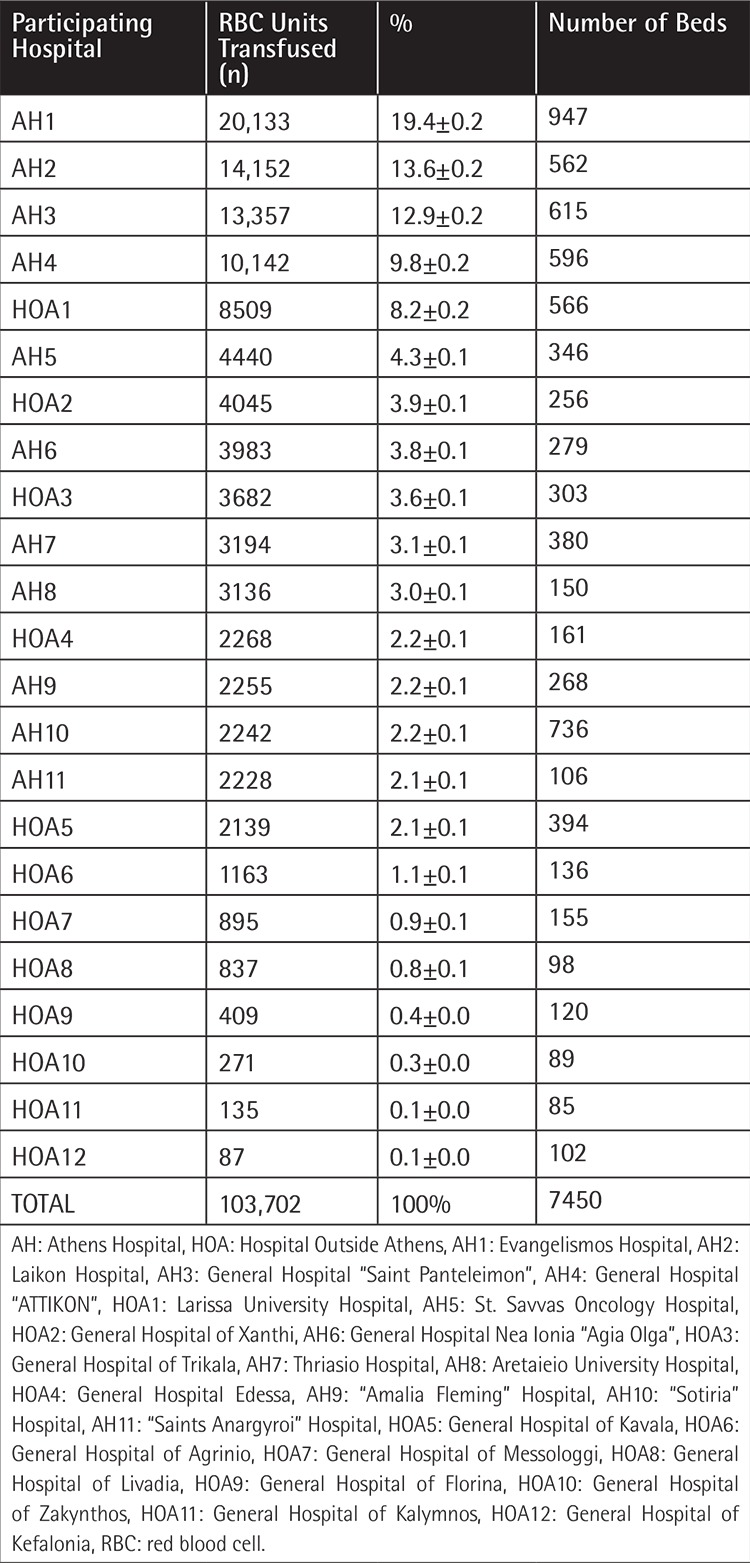
Number of red blood cell units transfused, percentages, and confidence intervals for the participating hospitals in declining order according to blood consumption.

**Table 2:**
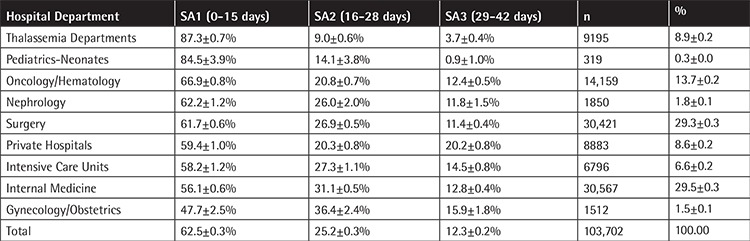
Percentages, totals, and confidence intervals for red blood cell consumption for the different hospital departments.

**Table 3:**
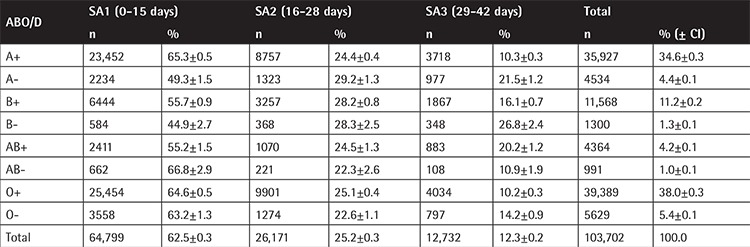
Number of red blood cell units, percentages, and confidence intervals according to ABO and RhD blood group for the three storage age groups (SA1, SA2, and SA3).

**Table 4:**
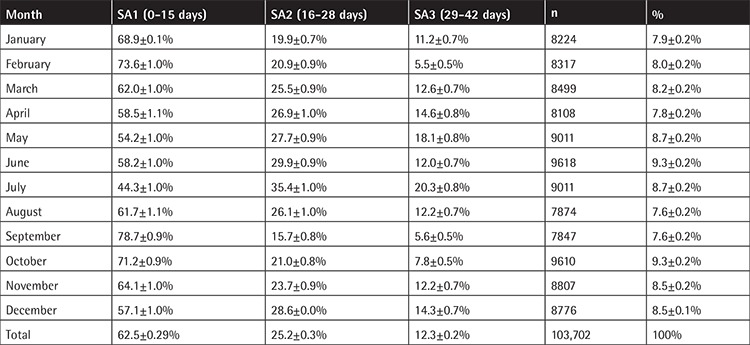
Red blood cell units transfused, percentages, and confidence intervals for each month during the study and the storage age groups (SA1, SA2, and SA3).
